# A School-Based Human Papillomavirus Vaccination Program in Barretos, Brazil: Final Results of a Demonstrative Study

**DOI:** 10.1371/journal.pone.0062647

**Published:** 2013-04-24

**Authors:** José Humberto Tavares Guerreiro Fregnani, André Lopes Carvalho, José Eluf-Neto, Karina de Cássia Braga Ribeiro, Larissa de Melo Kuil, Tauana Arcadepani da Silva, Silvia Lapola Rodrigues, Edmundo Carvalho Mauad, Adhemar Longatto-Filho, Luisa Lina Villa

**Affiliations:** 1 Research and Teaching Institute – Barretos Cancer Hospital (IEP – HCB), Barretos, Brazil; 2 National Institute of Science and Technology of the Diseases Associated to the Papillomavirus (INCT-HPV)/Santa Casa de São Paulo Medical School- FCMSCSP), São Paulo, Brazil; 3 Departament of Preventive Medicine – Faculdade de Medicina, Universidade de São Paulo (USP), São Paulo, Brazil; 4 Departament of Social Medicine – Santa Casa de São Paulo Medical School (FCMSCSP), São Paulo, Brazil; 5 Medical Research Laboratory 14– Medicine School of Universidade de São Paulo (USP), São Paulo, Brazil; The Catalan Institute of Oncology (ICO), Spain

## Abstract

**Introduction:**

The implementation of a public HPV vaccination program in several developing countries, especially in Latin America, is a great challenge for health care specialists.

**Aim:**

To evaluate the uptake and the three-dose completion rates of a school-based HPV vaccination program in Barretos (Brazil).

**Methods:**

The study included girls who were enrolled in public and private schools and who regularly attended the sixth and seventh grades of elementary school (mean age: 11.9 years). A meeting with the parents or guardians occurred approximately one week before the vaccination in order to explain the project and clarify the doubts. The quadrivalent vaccine was administered using the same schedule as in the product package (0–2–6 months). The school visits for regular vaccination occurred on previously scheduled dates. The vaccine was also made available at Barretos Cancer Hospital for the girls who could not be vaccinated on the day when the team visited the school.

**Results:**

Among the potential candidates for vaccination (n = 1,574), the parents or guardians of 1,513 girls (96.1%) responded to the invitation to participate in the study. A total of 1,389 parents or guardians agreed to participate in the program (acceptance rate = 91.8%). The main reason for refusing to participate in the vaccination program was fear of adverse events. The vaccine uptake rates for the first, second, and third doses were 87.5%, 86.3% and 85.0%, respectively. The three-dose completion rate was 97.2%.

**Conclusions:**

This demonstrative study achieved high rates of vaccination uptake and completion of three vaccine doses in children 10–16 years old from Brazil. The feasibility and success of an HPV vaccination program for adolescents in a developing country may depend on the integration between the public health and schooling systems.

## Introduction

Infection by the human papillomavirus (HPV) is the most common sexually transmitted disease [1], [2]. The data from the World Health Organization estimate that approximately 440 million people have genital HPV infection worldwide [3]. Cervical cancer is the most important disease that is caused by HPV and is a serious public health problem around the world. Cervical cancer is the third most common cancer among women worldwide. Approximately 530,000 new cases and 275,000 deaths due to the disease are reported annually [4]. It is estimated that, by 2030, the number of cervical cancer cases in the world will increase by approximately 50% [5].

The incidence and mortality rates of cervical cancer have decreased over the last 40 years in developed countries. However, this scenario has not occurred in most developing countries. The introduction of the Papanicolaou test as a secondary cervical cancer prevention strategy in the 1950 s caused a significant reduction in the incidence and mortality that was caused by the disease in most of Europe, North America, and Australia/New Zealand. In contrast, several developing countries did not demonstrate significant results due to low coverage of the Papanicolaou test, poor quality of the cytological exams, and difficult access to health services for the treatment of precursor lesions and cervical cancer [6]. These data support the need for incorporating new strategies and technologies to improve cervical cancer screening programs around the world.

The development of vaccines against HPV offers a viable approach for the primary prevention of cervical cancer. Clinical trials that have studied the quadrivalent and bivalent HPV vaccines have indicated that these vaccines are safe and effective for prevention of the precursor lesions of cervical carcinoma [7]–[13]. Although there are no published concrete data on the reduction of cervical cancer incidence and mortality rates after the HPV vaccine was implemented, these rates are expected to decline during the upcoming years. The data that were derived from the Australian HPV vaccination program support this prediction. After implementing the vaccination program, a rapid and marked reduction in the incidence of genital warts in young Australian women was observed [14]. More recently, Brotherton et al. [15] found, for the first time, a significant reduction in the incidence of high-grade abnormal cervical cytology in young women.

Since 2009, the World Health Organization has recommended that the HPV vaccine be included in national immunization programs [16]. In Brazil, the Ministry of Health approved the use of the quadrivalent and bivalent vaccines in August 2006 and February 2008, respectively. However, because of the cost and budget impact and the lack of a clear evidence of reduction in the incidence and mortality coefficients of cervical cancer, the vaccine had not been incorporated into a nationwide public immunization program in Brazil by mid-2013, being available only in private setting. Despite this issue, two papers have already shown that HPV vaccine is cost-effective in Brazil, especially in a high coverage rate scenario [17], [18].

The implementation of a public HPV vaccination program in low and middle income countries, especially in Latin America, is a great challenge for health care specialists. In addition to financial constraints, the ideal age group for vaccination, the best program compliance methods, and the duration of the immunization provided by the vaccine are under debate. Additionally, there is concern that the vaccine may interfere negatively in women’s participation to cervical cytology screening programs.

Although a number of studies on strategies for HPV delivery have been published worldwide [19]–[34], data from South America are scarce [35]. The main objective of this study was to evaluate the uptake and the three-dose completion rates of a school-based HPV vaccination strategy in a Brazilian city.

## Methods

This was a demonstrative study conducted in a group of adolescents residing in Barretos (SP), Brazil. Barretos is a rural town in the State of São Paulo in Southeastern of Brazil, which is located approximately 230 miles from the State capital (road distance). Barretos is an affluent region, and the economy is based on agriculture and the industrialization of meat both for domestic and export markets. This town has a population of approximately 112,000 inhabitants, 3% of which reside in rural areas [36].

This study was previously approved by the Research Ethics Committee of Barretos Cancer Hospital (Hospital de Câncer de Barretos – BCH, protocol number 291/2010) and registered at the National Health Institutes of the United States (NCT01159834). All guardians signed the informed consent on behalf of their girls.

### Study Population

**Figure 1:**
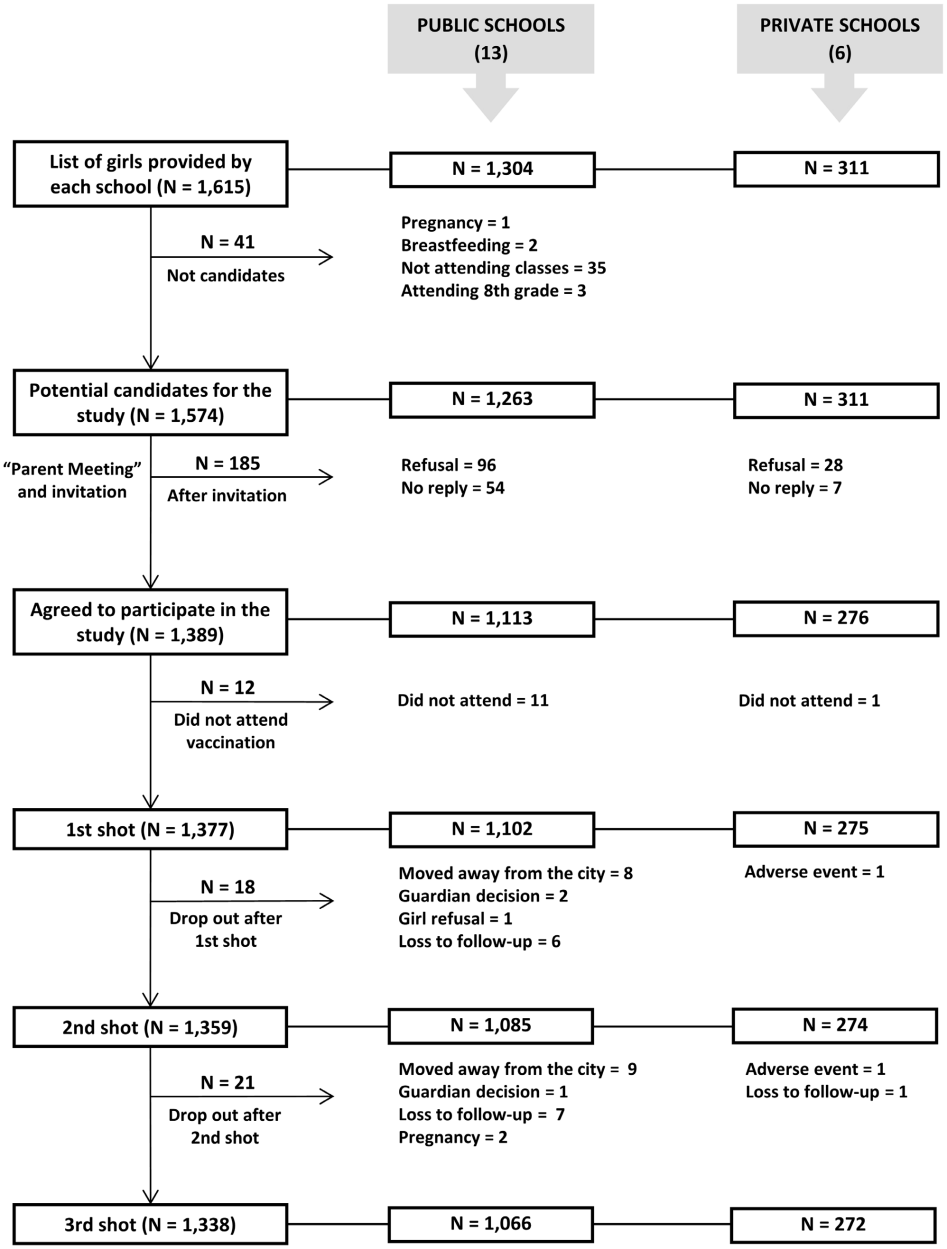
Distribution of girls throughout the study.

The study included girls who were enrolled in public and private schools and who regularly attended the sixth and seventh grades of elementary school. Pregnant girls, girls who had recently given birth and were breastfeeding and girls who were not attending classes despite being enrolled were excluded from the study. According to the inclusion criteria, 19 schools were candidates for the study (13 public schools and 6 private schools). Out of these schools, only one was located in rural area (27 girls). A total of 1,615 girls were identified by the schools, 41 of which were not eligible to participate in the study. Thus, a total of 1,574 girls were considered potential candidates for the study. Figure 1 shows the reasons for ineligibility and the distribution of girls throughout the study.

The characteristics of the parents and guardians of the girls who agreed to participate in the study are shown in Table 1. The age of the girls ranged from 10–16 years of age with a mean age of 11.9 years (SD = 1.0). There were no significant differences between the mean age of the public school students and that of the private school students (12.3 *vs.* 12.0; *P* = 0.268). Approximately 25% of the girls reported having had a boyfriend, and 2.6% (n = 33) of the girls stated having had sexual intercourse. The mean age of first sexual activity was 11.8 years (SD = 1.8, range: 8–15 years), and the number of sexual partners ranged from 1–3 (mean = 1.5, SD = 0.8). The rate of girls who had previous sexual intercourse was significantly higher in the public schools (3.2% *vs.* 0.4%; *P* = 0.012).

**Table 1:** Characteristics of the parents and guardians of the girls who agreed to participate in the vaccination program according to school type (n = 1,389).

Characteristic	Valid cases (*)	Description	Overall		Private school		Public school		P value
			N	(%)	N	(%)	N	(%)	
Guardian who gave authorization	1,182 (85.1%)	Mother/Father	1,073	(90.8%)	239	(94.8%)	834	(89.7%)	0.020
		Grandparents	48	(4.1%)	9	(3.6%)	39	(4.2%)	
		Aunt/Uncle	35	(3.0%)	4	(1.6%)	31	(3.3%)	
		Other	26	(2.2%)	0	(0.0%)	26	(2.8%)	
Age (years-old)	1,088 (78.3%)	Mean (SD)	38.1	(7.9)	39.8	(7.3)	37.7	(8.0)	<0.001
Family income (montly)	1,187 (85.5%)	≤US $250	268	(22.6%)	7	(2.8%)	261	(27.8%)	<0.001
		US $201–500	319	(26.9%)	21	(8.5%)	298	(31.7%)	
		US $501–1,000	368	(31.0%)	67	(27.0%)	301	(32.1%)	
		>US $1,000	232	(19.5%)	153	(61.7%)	79	(8.4%)	
Education (years of study)	1,203 (86.6%)	0–4 years	265	(22.0%)	5	(2.0%)	260	(27.3%)	<0.001
		5–8 years	236	(19.6%)	15	(6.0%)	221	(23.2%)	
		9–11 years	484	(40.3%)	111	(44.6%)	373	(39.1%)	
		>11 years	218	(18.1%)	118	(47.4%)	100	(10.5%)	
Religion	1,209 (87.0%)	None	41	(3.4%)	5	(2.0%)	36	(3.7%)	0.027
		Catholic	793	(65.6%)	180	(72.6%)	613	(63.8%)	
		Non-catholic	375	(31.0%)	63	(25.4%)	312	(32.5%)	
Race (skin color)	1,169 (84.2%)	White	649	(55.5%)	201	(82.4%)	448	(48.4%)	<0.001
		Non-white	520	(44.5%)	43	(17.6%)	477	(51.6%)	
Had you heard about the HPV vaccine?	1,170 (84.2%)	No	646	(55.2%)	111	(46.6%)	535	(57.4%)	0.003
		Yes	524	(44.8%)	127	(53.4%)	397	(42.6%)	
Area	1,389 (100.0%)	Urban	1,365	(98.3%)	276	(100.0%)	1,089	(97.8%)	0.008
		Rural	24	(1.7%)	0	(0.0%)	24	(2.2%)	

(*) Cases in which information was available for 1,389 parents and guardians who agreed to participate in the study.

### Pre-vaccination

**Figure 2:**
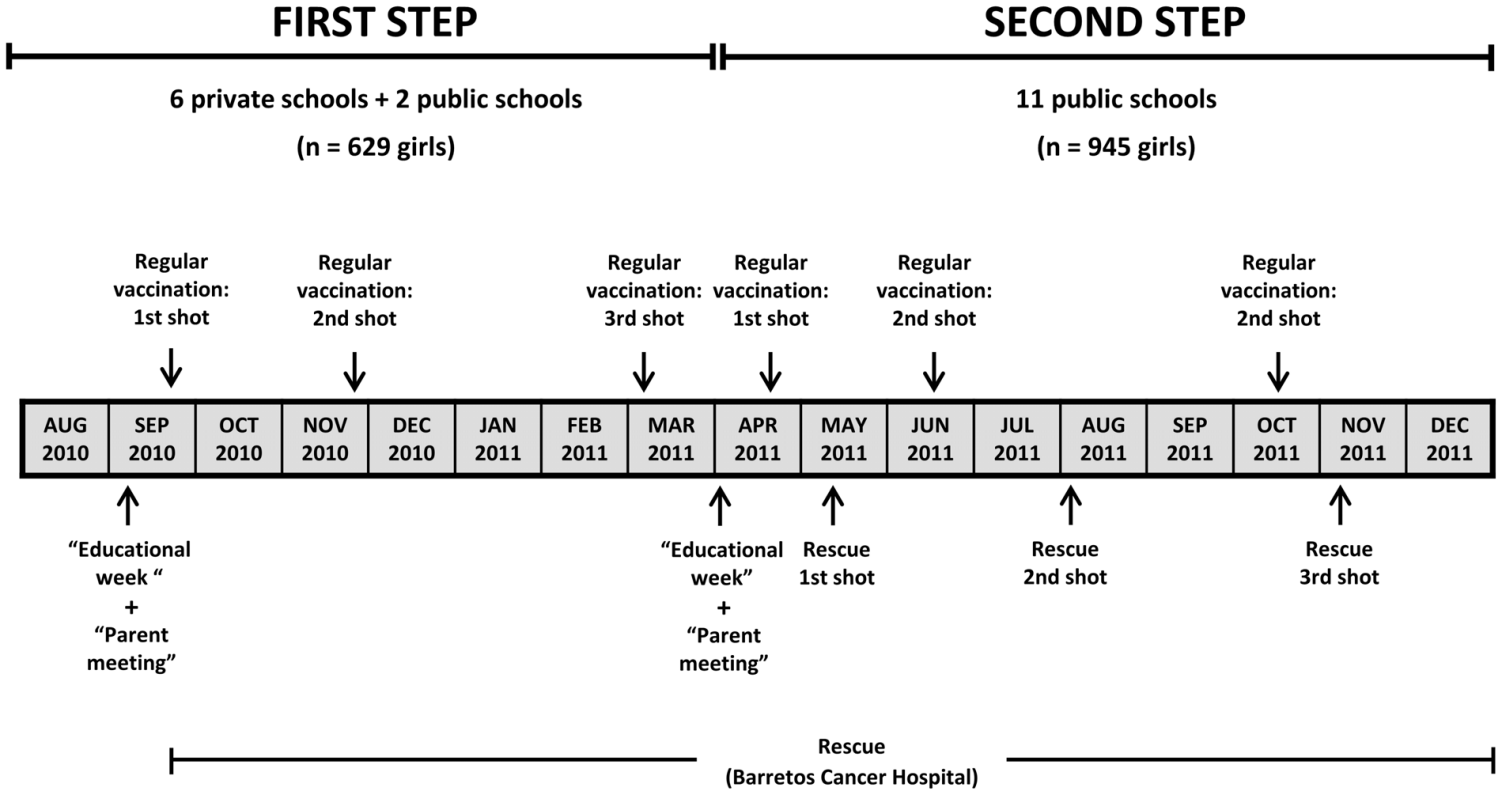
Design of the study (timeline).

Figure 2 summarizes the study. Due to logistic issues and the number of girls to be vaccinated, the study was divided into two steps: the first step included two public schools and six private schools (beginning in September 2010), and the second step included the other public schools (beginning in April 2011).

Approximately 30 days before initiating each of the two steps, the study was announced in the city through widespread advertising on billboards, TV shows and local radio stations, newspaper articles and the internet. Additionally, there was a meeting with the teachers and the principals of each school to clarify information about the study. During these meetings, the teachers and principals were asked to schedule an “Educational Week” approximately two weeks before the vaccination, during which the schools would work with students on school projects about HPV and the vaccine. During this week, the principals were asked to send written invitations to parents to come to the school on a scheduled date for a “Parent Meeting”. These invitations were sent to the parents and guardians via their daughters. During the first step of the study, schools were allowed to select the educational strategies for the “Educational Week” with the children. However, the schools offered different levels of commitment, which was lower in several public schools when compared to the private schools. During the second step of the vaccination, a visit from a study nurse was included in the “Educational Week” at each school. On this occasion, the nurse met with the girls and discussed sexual education, sexually transmitted diseases, HPV and the vaccine. During this meeting with the girls, the nurse stressed the importance of ensuring that parents attended the “Parent Meeting.”

The “Parent Meeting” occurred approximately one week after the “Educational Week” and approximately one week before the vaccination was administered in the schools. A physician and a nurse (related to the study) visited each school to present information about the study. During these meetings, they clarified doubts about the project. The parents (or legal guardians) filled out a questionnaire and those who agreed gave their informed consent to vaccinate their child and. Those who could not read were assisted by one of the teachers or professionals who were conducting the study. The parents who did not attend the meeting, but who expressed interest in the vaccination, could later enroll in the study.

### Vaccination

The quadrivalent vaccine (Gardasil™ - Human Papillomavirus Quadrivalent - Types 6, 11, 16, and 18– Recombinant Vaccine, Merck & Co., Inc.) was used in the study and the proposed schedule was the same to that in the product package insert (the second dose two months after the first dose and the third dose six months after the first dose). An alternative schedule was needed (0–1–4 months) for some girls who were later enrolled.

The school visits for regular vaccination occurred on previously scheduled dates. Three nurses and an assistant from BCH composed the school vaccination study team. On the day of the visit, each school provided a classroom where the vaccine could be administered. Girls were taken out of class to receive the vaccine. Just before the vaccination, they completed a questionnaire with demographic and sexual behavior information (self-completed questionnaire). During this time, the girls were informed that the questionnaire responses were confidential. Afterwards, the girls were referred to the vaccination room in small groups of two or three girls to prevent long lines outside of the classroom. The purpose of this measure was to prevent the so-called “*Mass Psychogenic Illness*”, which consists of an outbreak of symptoms that are similar among a group of vaccine recipients because the vaccinees witnessed the reactions of the other vaccinees [19], [20]. The girls who had a fever, cold symptoms, or an infection on the day of the vaccination were instructed to postpone the vaccine and visit BCH at a later date to receive the vaccine. After vaccination, the girls remained seated, were observed for 15 minutes and were then allowed to resume their activities.

The vaccine was also made available at BHC for the girls who could not be vaccinated on the day when the team visited the school (rescue vaccination/mop-up vaccination). During the first step of the study, after the first visit to the schools, the study team contacted the girls who missed the opportunity for vaccination at the school by phone call. During the second step of the study, there was a second round of vaccination at the schools a few weeks after the initial scheduled visit, in addition to rescue vaccination at BCH. Immediately after the regular vaccination at the schools, the principals were asked to inform parents or guardians in writing that the vaccination team would return to the school for a second round of vaccination (rescue vaccination at schools) within two to six weeks. During the second step, phone calls were made only to the girls who were not vaccinated during the second round of vaccination.

### Statistical Analysis

In this study, the statistics were calculated according to the following definitions:

- Vaccine acceptance rate: the relative proportion of girls whose parents or guardians agreed to participate in the study to the total eligible girls (n = 1,574).
- Vaccine uptake rate: the relative proportion of vaccinated girls to the total number of eligible girls (n = 1,574). This rate was calculated separately for the first, second, and third doses.
- Three-dose completion rate: the relative proportion of girls who completed vaccination to the total number of girls who only received the first dose (n = 1,377).
- Need for rescue vaccination: the relative proportion of girls who needed rescue vaccination (mop-up) at BCH or during the second visit to the schools for a vaccine update to the total number of girls.

The casuistic was characterized by descriptive statistics. Categorical variables were compared using the chi-squared test or Fisher’s exact test, depending on the expected values in the contingency tables. The mean values of the numeric variables were compared using Student’s *t*-test. The significance level was set at 5%, and all of the tests were two-tailed. MedCalc version 11 was used to calculate the confidence intervals of the proportions (MedCalc Software™). The other statistical analyses were performed using SPSS version 19.0 software (IBM/SPSS, Inc.).

This study was conducted by the investigator with institutional support from BCH, the National Institute of Science and Technology of Papillomavirus Diseases (INCT-HPV), and Merck & Co., Inc. who provided the vaccine by Merck Investigator Studies Program (MISP). The company had no role in the study design, data collection, analysis, interpretation of the results, writing of the report, or the decision to submit the paper for publication. All of the statistics were impartially performed by the Center for Researcher Support of BCH.

## Results

### Advertising Methods

The parents and guardians claimed that they were informed about the study through the following advertising media: invitation prepared by the school (80.5%; 95% CI: 75.7%–85.6%), local media (16.8%; 95% CI: 14.7%–19.2%), medical professionals (1.8%; 95% CI: 1.1%–2.7%) and other media (1.3%; 95% CI: 0.8%–2.1%). There were no significant differences between the methods by which the guardians were informed about the vaccination and school type.

### Vaccine Acceptance and Reasons for Refusal

Among the potential candidates for vaccination (n = 1,574), the parents and guardians of 1,513 girls (96.1%; 95% CI: 91.3%–100.0%) responded to the invitation to participate in the study. A total of 1,389 parents and guardians agreed to participate in the study.

The vaccine acceptance rate was 88.2% (95% CI: 83.7%–93.0%). There were no significant differences in the acceptance rates between public and private schools (Table 2). Regarding the school location, there was no difference in the acceptance rate according to rural and urban areas (96.0% *vs*. 91.7%, P = 0.716).

**Table 2:** Vaccination statistics according to school type.

Indicator	Description	Overall		Private school		Public school		P value
		(Eligible: N = 1,574)		(Eligible: N = 311)		(Eligible: N = 1,263)		
		N	(%)	N	(%)	N	(%)	
**Vaccine acceptance**	Yes	1,389	(88.2%)	276	(88.7%)	1,113	(88.1%)	0.836
**Vaccine uptake**	First dose	1,377	(87.5%)	275	(88.4%)	1102	(87.3%)	0.576
	Second dose	1,359	(86.3%)	274	(88.1%)	1,085	(85.9%)	0.312
	Third dose	1,338	(85.0%)	272	(87.5%)	1066	(84.4%)	0.185
**Three-dose completion**	Yes	1,338	(97.2%)	272	(98.9%)	1,066	(96.7%)	0.052
**Rescue vaccination**	First dose	279	(20.3%)	38	(13.8%)	241	(21.9%)	0.003
	Second dose	357	(26.3%)	50	(18.2%)	307	(28.3%)	0.001
	Third dose	291	(21.7%)	50	(18.4%)	241	(22.6%)	0.132

The parents and guardians of 124 girls expressly refused to participate in the study (Table 3). There was a larger proportion of parents and guardians of girls in private school who refused the study due to incorrect information about the vaccine (17.9% *vs.* 3.1%; *P* = 0.014) or medical advice to not allow the girls to receive the vaccination (14.3% *vs.* 3.1%; *P* = 0.045). There were no differences between public and private school according to the other reasons that were reported for refusal.

**Table 3:** Reasons given by the parents and guardians for refusing to participate in the vaccination program.

Reasons for refusing vaccination (*)	Overall		Private school		Public school		P value
	(n = 124 answers)		(n = 28 answers)		(n = 96 answers)		
	N	(%)	N	(%)	N	(%)	
Fear of adverse events	34	(27.4%)	8	(28.6%)	26	(27.1%)	0.877
Undisclosed personal reason	25	(20.2%)	4	(14.3%)	21	(21.9%)	0.378
The girl does not want to get the vaccine shot	18	(14.5%)	1	(3.6%)	17	(17.7%)	0.072
The girl is too young (age)	12	(9.7%)	5	(17.9%)	7	(7.3%)	0.140
Girl has a health problem	12	(9.7%)	4	(14.3%)	8	(8.3%)	0.466
Belief that the vaccine is not necessary	11	(8.9%)	3	(10.7%)	8	(8.3%)	0.710
Incorrect information about the vaccine	8	(6.5%)	5	(17.9%)	3	(3.1%)	0.014
Physician advised against it (pediatrician/gynecologist)	7	(5.6%)	4	(14.3%)	3	(3.1%)	0.045
Does not want to participate in a research study	5	(4.0%)	0	(0.0%)	5	(5.2%)	0.587
No trust in vaccine efficacy	3	(2.4%)	1	(3.6%)	2	(2.1%)	0.539
No knowledge of the vaccine	3	(2.4%)	0	(0.0%)	3	(3.1%)	1.000
Difficulties travelling to the hospital to get the vaccine	2	(1.6%)	0	(0.0%)	2	(2.1%)	1.000

(*) Responders could report more than one reason.

### Vaccine Uptake Rates

The vaccine uptake rates for the first, second, and third doses were 87.5% (95% CI: 82.9%–92.2%), 86.3% (95% CI: 81.8%–91.1%), and 85.0% (95% CI: 80.5%–89.7%), respectively. There were no statistically significant differences between uptake rates of the public and private schools (Table 2). Schools sited in rural and urban areas had similar uptake rates for the first (88.9% vs. 87.5%; P = 1.000), second (88.9% vs. 86.3%; P = 1.000) and third doses (85.2% vs. 85.0%; P = 1.000).

### Three-dose Completion Rate and Reasons for Leaving the Study

Of the girls who received the first dose, a total of 1,338 remained in the study until the third dose, which represents a three-dose completion rate of 97.2% (95% CI: 92.0%–100.0%). This rate was slightly lower in the girls who attended public school (96.7% *vs.* 98.9%; *P* = 0.052) when the type of school was compared (Table 2). No difference in the three-dose completion rate was observed regarding rural vs. urban areas (95.8% vs. 97.2%; P = 0.501).

The reasons for the discontinuation of the vaccination were as follows: moved away from the city (17 girls who attended public school), loss to follow-up (16 girls who attended public school and one who attended private school), the decision of the parents or guardians with no clear justification (3 girls who attended public school), girl refusal to continue the vaccination (1 girl who attended public school) and pregnancy (2 girls who attended public school). Two girls in private school discontinued the vaccination due to adverse events that were reported by family members, none of which were classified as serious by the medical study team.

### Rescue Vaccination

Rescue vaccination was necessary in 279 cases for the first dose (20.3%; 95% CI: 17.9%–22.8%), 357 cases for the second dose (26.3%; 95% CI = 23.6%–29.1%) and 291 cases for the third dose (21.7%; 95% CI = 19.3%–24.4%). There was a difference in the need for rescue vaccination between the schools for the first and second doses of the vaccine, and this need was significantly higher in public schools: first dose (21.9% *vs.* 13.8%; *P* = 0.003) and second dose (28.3% *vs.* 18.2%; *P* = 0.001). The difference was not statistically significant for the third dose, although the need for rescue vaccination was higher in public schools (22.6% *vs.* 18.4%; *P* = 0.139) (Table 2).

### Adverse Events

The adverse events that were observed or reported in this study were as follows: lipothymia accompanied by skin paleness and/or sudoresis (n = 11), fever (n = 7), vomiting and nausea (n = 5), pain and edema at the injection site (n = 5), transient tremors (n = 3), facial edema (n = 2), skin rash (n = 2), headache (n = 2), facial flushing (n = 1), skin spots (n = 1) and sleepiness (n = 1). No serious adverse events were reported during the study.

## Discussion

Australia was the first country to establish a national public HPV vaccination program. In April 2007, the Australian government started a school-based vaccination program that included girls between the ages of 12 and 13. Two other catch-up programs were conducted in parallel until December 2009. One program was school-based and the other program was community-based. Women up to 17 years of age were vaccinated in schools, and older women or those who missed the vaccination at school could be vaccinated at community health centers (general practitioners, university health services, women health centers, and family planning services) [20]. The results of the program in Australia from April 2007 to December 2009 were recently published by the Australian government. The observed coverage rate was high in the school-based program. The coverage rate for the third dose was approximately 75% in girls between the ages of 12 and 15 years. However, these rates were lower in women over 18 years of age and in women who were vaccinated in a community-based program (38% for women 18–19 years of age and 30% for women 20–26 years of age) [20], [21].

The Brazilian Immunization Program, with approximately 40 years of tradition, is internationally recognized for its quality and high coverage rates, especially in childhood vaccination. Official government data indicate coverage rates over 90% for the most important childhood vaccines [37]. However, the scenario is not the same for adolescents and adults. Vaccination in adolescents, especially in older age groups, is complicated. In the United States, for instance, the overall vaccine coverage rate in 2009 among adolescents between the ages of 13 and 17 years was approximately 50% for males and 33% for females [38].

This study obtained relevant results related to the indicators of HPV vaccination in adolescent Brazilian girls with a school-based vaccination strategy. To the best of our knowledge, this is the first HPV vaccine study of this type in Brazil. Favorable results were also reported in other countries that used a school-based approach, even in low resource setting. Singh et al. described an initial experience in Nepal using a school-based strategy to vaccinate 1,096 women aged 10–26 years from 17 secondary schools. The uptake rates were expressively high for the first (100%), second (99.5%) and third (99.3%) doses [22]. LaMontagne et al. reported significant coverage rates in a study that evaluated school-based HPV vaccination in adolescent girls (mostly between the ages of 9 and 14 years) in Peru, Uganda, Vietnam and India. The coverage rates were 82.6% (2008) in Peru, 90.5% (2008) and 88.9% (2009) in Uganda and 83.0% (2008–2009) and 96.1% (2009–2010) in Vietnam. In India, the programs were mixed, involving school-based strategies, health centers, and campaigns in the community. The rates ranged from 68.4%–87.8%, depending on the target population (urban, rural, or tribal) [23]. Ladner et al. recently published the results of eight HPV vaccination programs conducted in seven low-income countries (Bhutan, Bolivia, Cambodia, Cameroon, Haiti, Lesotho, and Nepal) through the Gardasil Access Program involving more than 87,000 girls (depending on the program, age ranged from 9 to 18). Three programs adopted a school-based strategy, two used health facility-based approach and 3 used combined strategies (schools and health facilities). The health facility model had the worst vaccine coverage rate (77.1%). The other strategies involving school-based approaches had coverage rates above 90% [24]. Two different school-based delivery strategies (age-based versus class-based) were assessed by Watson-Jones et al. in a cluster-randomized trial of HPV vaccination conducted Tanzania. The study included more than 5,000 girls from 134 primary schools (median age: 13 years). The overall coverage rates for the first, second and third doses were respectively 84.7%, 81.4% and 76.1%. The class-based delivery had higher coverage rate than the age-based strategy [25].

In contrast, the literature has systematically indicated low coverage or uptake rates for the HPV vaccine in non-school-based programs [20], [26]–[31], [33]. In Australia, vaccination in the community did not achieve results that were as significant as those achieved with the school-based strategy [20], [21]. In the United States where the vaccination program is not school-based, the HPV vaccine coverage rate in 2011 among girls was only 53% for one dose (or more) and 35% for 3 doses [33]. Additionally, the data that were derived from several countries in Europe revealed low coverage or uptake rates of the first dose of the vaccine in strategies that did not involve schools: the Netherlands (adolescents between 13 and 16 years of age: 49.9%) [26], Italy (Desio and Sesto San Giovanni districts, adolescents 12 years of age: 55.3%) [27], France (maximum coverage in adolescents 15 years of age: 52.5%) [29] and Belgium (adolescents between 12 and 15 years of age: 44%) [30]. However, a few non-school-based HPV vaccination programs achieved significant coverage or uptake rates, such as in Denmark, Spain and Mexico [32], [34], [35]. Data from the program in Denmark indicated a uptake rate of 85% for the first dose in 12-year-old girls [32]. In Spain, the vaccination program for girls 11–14 years of age varied according to the region. The coverage rate was 70.1% in regions where vaccination was performed in health centers, whereas the rate was higher in regions that adopted the school-based strategy (84.2%) [34]. In Mexico, the primary HPV immunization program did not include all of the cities in the country but only those with a lower human development index. This program achieved a coverage rate of 85% for the first dose in girls 9–12 years of age (2009). In 2011, the Mexican government expanded the vaccination program to the entire country, including a school-based vaccination strategy for 9-year-old girls [35]. In Latin America, the data regarding the HPV vaccine acceptance and coverage rates remain scarce. Up to mid-2012, the only countries in Latin America that had incorporated the vaccine were Mexico, Panama and Argentina [35], [39].

The vaccination strategy that was adopted in this study may not be easily reproducible on a large scale in Brazil. The current Brazilian public vaccination process is focused mainly on government health units (Basic Health Units, Reference Centers for Special Immunobiologicals), and national campaigns. There are no programs that are dedicated to regular vaccination in schools. It is likely that the Brazilian government may not be willing to change the immunization system because the current childhood vaccination program has achieved high coverage levels for the vaccines that are available in the public system [37]. Despite this scenario, the results of this study suggest that the integration of health and education systems is an important step to satisfy appropriate vaccine indicators. In addition, a combined strategy, involving schools and public health facilities can be interesting, as suggested in the study recently published by Ladner et al [24]. Because of the continental size and marked economic contrasts between certain regions in Brazil, several vaccination strategies that are adapted to regional characteristics would be more successful than a single immunization program against HPV. Vaccination in schools is one of many strategies that could be adopted. Nevertheless, further studies are needed in order to support this strategy, especially those involving cost-effectiveness analyses.

Although the HPV vaccine has been approved for use in Brazil for approximately 5 years, approximately half of the parents and guardians of the girls in this study had no knowledge of the vaccine before the study. The percentage of parents and guardians who knew about the vaccine was significantly higher among those with better economic and educational backgrounds (private school). These data suggest that a HPV vaccination program, even when performed in a population with a high level of education, does not guarantee a sufficient level of knowledge about the vaccine without appropriate advertising. The significant results that were obtained in this study can be attributed to a primarily school-based advertising strategy and not to previous knowledge about the vaccine.

The assessment of how the parents or guardians were initially informed about the vaccination indicates that the information disseminated by the schools had a significant role in the vaccine indicators. When the girls were adequately informed at school, they actively conveyed information about the vaccine to their parents or guardians at home. The local media is usually considered one of the main means of communication. However, the media had a less important role in advertising this vaccination initiative. These data suggest that the participation of schools in an HPV vaccination program is important to adequately disseminate information about the vaccine and increase vaccine acceptance.

Despite the high uptake and completion rates that were obtained in this study, these rates may not be as significant in a real public health scenario (large scale vaccination). Three aspects must be discussed regarding this issue. 1) The first aspect is that BCH is known in Brazil to provide excellent health services to the community, especially in cancer prevention. Thus, any programs that are initiated and conducted by BCH have high credibility with the local community. This credibility most likely contributed to the high acceptance of the vaccination program in Barretos; 2) A second aspect that may have contributed to the results that were obtained in this study was the experimental nature of this vaccination program. Because this program was part of a research study, active contact was sought with the girls who missed the opportunity to be vaccinated at school. In a real population scenario that involves millions of adolescents, an active search system would not be feasible. Approximately all of the vaccination programs in Brazil are based on spontaneous demand and campaigns. In the second step of this study, we included vaccination at BCH and a second round of vaccination at the schools as a vaccine rescue strategy. This strategy significantly reduced the number of phone calls to the adolescents who missed the first round of vaccination. If this active search had not been performed, a decrease of approximately 20–25 percentage points in the uptake rate would have occurred. This decrease in the uptake was more relevant among the girls who attended public schools whose family incomes were low. The uptake rate of the girls in public school who completed three doses was high. However, the rate was still lower when compared to the rate of the girls in private school. This finding should be taken into account when planning and implementing a school-based vaccination system. To obtain high levels of vaccine coverage and completion, strategies should be adopted to ensure the opportunity for mopping-up, especially for adolescents of lower socioeconomic levels; 3) A third aspect that may have led to the expressive results was the fact that the majority of the schools in this study were located in urban areas and, even the single school sited in the rural zone, had easy access. The results achieved by this study may not be broadly extrapolated for other places in Brazil, especially for those remote regions with limited access to the schools located in rural area.

The data from the Brazilian hepatitis B vaccination program in adolescents could be used to estimate the HPV vaccination coverage for this age group in Brazil. According to the Brazilian government, the hepatitis B vaccine coverage rate (third dose) for adolescents 11–14 years of age was approximately 92%. However, this rate decreased to 60% among adolescents 15–19 years of age [40]. These data suggest that an HPV vaccination program in Brazil could benefit from being a school-based program, especially involving young adolescents. The vaccine coverage rate is an important issue not only to estimate the level of protection offered to a population, but also related to the cost-effectiveness aspect. At least two studies observed a profound impact of the coverage rate on the cost-effectiveness of HPV vaccine in Brazil [17], [18].

In this study, approximately 10% of the parents and guardians refused to participate in the vaccination program. The experimental nature of the study caused a higher refusal rate among parents and guardians, especially when the girls attended private school. After reading the informed consent form, several parents and guardians refused the program due to fear of vaccine adverse events, which were described in the document. Other parents and guardians were worried about participating in a research study and unduly believed that the girls would be treated as “laboratory guinea pigs.” It is likely that the refusal rate would be lower in a real population scenario. However, there will always be a part of the population that will not agree to participate in a vaccination program. This study verified that many of the reasons for refusal were related to insufficient clarification about the vaccine, such as the following: the belief that the girl was too young to be vaccinated, the belief that the vaccination was not necessary, a lack of trust in the efficacy of the vaccine, and a lack of knowledge about the existence of the vaccine. All of these reasons could be accounted for if appropriate information was provided and the HPV vaccine was included in public vaccination programs. Although the reasons for refusing the vaccination have been well characterized in the study (as shown in table 3), it is not possible to know the determinant factors involved in the guardianś decision to participate in the protocol because none of the guardians who refused was willing to answer the demographic questionnaire. Only the guardianś information who agreed to participate in the study was available, making the analysis of those determinant factors impossible.

### Conclusions

This demonstrative study is the first to evaluate a school-based HPV vaccination program in Brazil. The vaccine uptake rates were high and similar between public and private schools. The three-dose completion rate was slightly lower among those girls who attended public schools, but still high. Nevertheless, further studies, including cost-effectiveness analysis, are needed in order to define the best vaccine delivery strategy in Brazil.
